# RDML-Ninja and RDMLdb for standardized exchange of qPCR data

**DOI:** 10.1186/s12859-015-0637-6

**Published:** 2015-06-20

**Authors:** Jan M. Ruijter, Steve Lefever, Jasper Anckaert, Jan Hellemans, Michael W. Pfaffl, Vladimir Benes, Stephen A. Bustin, Jo Vandesompele, Andreas Untergasser, on behalf of the RDML consortium

**Affiliations:** Department of Anatomy, Embryology & Physiology, Academic Medical Center, Amsterdam, 1100AZ Netherlands; Center for Medical Genetics, Ghent University, Gent, B-9000 Belgium; Bioinformatics Institute Ghent, Ghent University, Ghent, B-9000 Belgium; Biogazelle, Zwijnaarde, 9052 Belgium; Physiology, Center of Life and Food Sciences, Technical University of Munich, Freising Weihenstephan, Munich, 85354 Germany; Genomics Core Facility, European Molecular Biology Laboratory, Heidelberg, 69117 Germany; Postgraduate Medical Institute, Anglia Ruskin University, Chelmsford, CM1 1SQ UK; Center of Molecular Biology (ZMBH), Heidelberg University, Im Neuenheimer Feld 282, Heidelberg, 69120 Germany

**Keywords:** qPCR, RDML, MIQE, RDML-Ninja, RDMLdb

## Abstract

**Background:**

The universal qPCR data exchange file format RDML is today well accepted by the scientific community, part of the MIQE guidelines and implemented in many qPCR instruments. With the increased use of RDML new challenges emerge. The flexibility of the RDML format resulted in some implementations that did not meet the expectations of the consortium in the level of support or the use of elements.

**Results:**

In the current RDML version 1.2 the description of the elements was sharpened. The open source editor RDML-Ninja was released (http://sourceforge.net/projects/qpcr-ninja/). RDML-Ninja allows to visualize, edit and validate RDML files and thus clarifies the use of RDML elements. Furthermore RDML-Ninja serves as reference implementation for RDML and enables migration between RDML versions independent of the instrument software. The database RDMLdb will serve as an online repository for RDML files and facilitate the exchange of RDML data (http://www.rdmldb.org). Authors can upload their RDML files and reference them in publications by the unique identifier provided by RDMLdb. The MIQE guidelines propose a rich set of information required to document each qPCR run. RDML provides the vehicle to store and maintain this information and current development aims at further integration of MIQE requirements into the RDML format.

**Conclusions:**

The editor RDML-Ninja and the database RDMLdb enable scientists to evaluate and exchange qPCR data in the instrument-independent RDML format. We are confident that this infrastructure will build the foundation for standardized qPCR data exchange among scientists, research groups, and during publication.

## Background

Real-time quantitative PCR (qPCR) is a powerful method for accurate measurement of nucleic acid concentrations. qPCR instruments collect a large set of data during each run, which provides the basis for quantification and PCR product validation. Further, the user has assembled data on biomaterials, targets and qPCR assays that are essential in determining data quality, downstream statistical analysis and independent replication; target and sample names can be entered into the qPCR instrument software to be used in built-in data analysis routines. An instrument-independent format to store and exchange this collection of data was published in 2009 as the first version of Real-time PCR Data Markup Language (RDML) [1]. Since then, several journals have endorsed and advocated the use of RDML (e.g. Clinical Chemistry, Nucleic Acids Research, BioMed Central series of journals, etc.; see News section on RDML website). Of practical relevance, Bio-Rad (CFX96 and CFX384), Life Technologies (StepOne, ViiA7 and QuantStudio), Qiagen (Rotor-Gene Q) and Roche (LightCycler96) implemented RDML export capabilities into their instrument's software. Furthermore, third party software with RDML data file import and/or export functionality, such as LinRegPCR ([2]; http://LinRegPCR.nl), qbase + ([3]; http://www.qbaseplus.com) and an RDML R package, were developed to enable (part of the) data-analysis workflow, such as raw data quality control, amplification curve analysis, normalization and statistical analysis independent of the qPCR machine software (see [4] and [5] for an overview of qPCR data analysis tools). As a design choice, many elements in the RDML data tree are optional and documentation fields are present in several places. This choice allows the flexible use of RDML even in non-qPCR applications. Primer3plus is an example of this intended use, where primer and amplicon sequences can be exported as RDML files containing only the “target” elements of the RDML format [6]. On the down side, this flexibility resulted in cases where the level of RDML support did not fully meet the expectations and the original intentions of the RDML consortium. Some qPCR instrument software export only a small set of the collected data and ignore available RDML elements, or the data are stored in different elements or in different formats than intended. However, due to the flexibility of RDML, the software would still export valid files in these cases. The RDML consortium aims to overcome these issues by clarification of the RDML elements that led to misinterpretation and by supporting the software developers with tools to create, analyze and validate RDML files.

One of the intentions for creating RDML was to facilitate the exchange of raw fluorescence data free of smoothing or baseline subtraction. Raw data are the holy grail of qPCR analysis as they allow quality control, evaluation of the validity of conclusions and, if new methods or statistical analysis tools become available, re-evaluation of previously published results. Furthermore raw data open the door for meta-analysis of published qPCR experiments without any bias from the original analysis. Today the RDML format offers instrument independency and free and straightforward data exchange, but publications with RDML files as supplemental data are still the exception. Furthermore, large experimental sets result in RDML files of significant size, and journals may prefer to not store these files on their website. A central repository dedicated to RDML files offers a better solution for easy exchange. Authors upload their RDML files into this database and provide the matching IDs in the article, as is customarily done for microarray and RNA-sequencing data (through e.g. Gene Expression Omnibus, Sequence Read Archive, or European Nucleotide Archive).

In this paper we describe the evolution of RDML up to version 1.2, present the data file editor RDML-Ninja and the database RDMLdb, a database dedicated to the storage and exchange of RDML files.

## Implementation

### RDML version development

RDML files are compressed text files containing an XML-based hierarchical tree with elements for experimenters, documentations, dyes, samples, targets, cycling conditions and experiments at the top level. Each element contains various sub-elements. The experiment element contains run elements, each containing the set of reactions in the run with the fluorescence data, baseline values, quantification threshold and observed Cq value as sub-elements. A reaction element refers to a sample element and a target element by their unique IDs.

The release of a new RDML version is coordinated by the RDML consortium (www.rdml.org). Any researcher, programmer or data-analyst in academia as well as industry can join the consortium free of charge and can participate in the development of RDML. Suggestions are evaluated, discussed in the consortium and implemented by the RDML core group. Based on the community feedback, the new version is created and released. The consortium aims for an abstracted design that can be used with the majority of instruments and software packages available and thereby balances the interests of the different instrument providers and qPCR users.

### Software organisation of RDML-Ninja

RDML-Ninja is an editor that allows the researcher to view and to modify the contents of RDML data files. The RDML data is stored as a XML tree structure from where entries are read on demand and placed in the respective elements of the graphic interface ready for user interaction. For elements such as “sample” or “target”, RDML files allow one up to an unlimited number of entries. In such a case, the user first chooses one element from a list with all elements to access the sub-elements of this selected element (analogous to opening the branch of a folder tree in a computer operating system). The position of the chosen element in the XML tree is saved in memory upon selection. If the user decides to modify an entry via the graphic interface, the data can thus be written back to the corresponding position of the XML tree structure. Prior to modification of the XML tree, the validity of the user input is checked and the operation will proceed only when the input is valid. The majority of data collected by the instruments are stored within the “react” elements and their sub-elements. RDML-Ninja enables to view, but not modify, this part of the XML tree in a table view reconstructing the plate format. Furthermore, the collected fluorescence values can be plotted as amplification curves or melt curves and exported into the Scalable Vector Graphics (SVG) format for further use in presentations or publications.

The platform independence of RDML-Ninja as well as its native look was achieved by the use of the Qt cross-platform application framework (Qt; http://www.qt-project.org). Precompiled executable programs are available for Microsoft Windows and Apple OS X platform (http://sourceforge.net/projects/qpcr-ninja/). Because of the design of the Qt framework, the support can be easily extended to other platforms if required. The application code was written in C++ making heavy use of Qt functionality, not only to display the graphical interface but also to store XML data or draw SVG graphics. The software is freely available for commercial and academic use under GNU General Public licence (GPL).

### Overview of RDMLdb

RDMLdb is an online database for exchanging RDML files (http://www.rdmldb.org). Users access the database via an interactive web interface. The database stores the original RDML files as well as key information extracted from these files required to index the database. Users can query the database based on the generated index to find a specific RDML file.

The majority of the web interface scripts of RDMLdb are written in PHP 5.3.10 and use the JavaScript library jQuery 1.10.2 to enhance usability by providing auto-completion of search fields and form validation. At time of upload, the user only provides an RDML file. This file is parsed using a Perl 5.14.2 script to extract the meta information required to create a record such as version, target, sample and experiment description. The record is complemented with a unique ID, some additional fields such as the PubMed ID, the email address of the uploading user as well as the date of the upload. The meta information record and the original file are stored in the database. The database functionality was provided by using MongoDB 2.6.7 (http://www.mongodb.org) because its NoSQL structure allows varying number of fields in each record and it enables the storage of entire files within the database using a gridFS system. Users may query the database using the unique ID or search the above listed fields and then download the associated RDML files.

## Results

### RDML version development 1.1 and 1.2

Since the release of RDML version 1.0 in 2009 [1], the RDML standard was updated twice with the latest version 1.2 being released in Autumn 2014. The major change in version 1.1 was a complete redesign of the plate setup. In the initial RDML version 1.0, all possible plate setups were predefined and therefore each new instrument making use of a new plate type required an update of the RDML standard. To overcome this instrument dependency, from version 1.1 onwards, the plate setup has been described by providing the number of available reactions in two dimensions. The identification of a single reaction was changed from the letter (row) + number (column) format to a number only format based on the reaction position in the two-dimensional matrix. Additionally, the handling of the dye element was redesigned. Being originally an optional element, the plate setup of multiplex reactions could not be reconstructed without dye information. To avoid ambiguous situations, the dye element is no longer optional and all dyes must be registered at top level.

RDML version 1.2 addresses the need to classify samples into groups, in order to facilitate downstream statistical analysis. Therefore, an annotation element was introduced containing a property and a value string as sub elements. Each defined sample may have several of these annotation elements. We envision the use, for example, in a mouse experiment, where a first annotation element could have the property “gender” with the values “male” or “female” and a second element could have the property “treatment” with the values “control”,“condition1” or “condition2”. The free string format for properties and values allows flexible tagging of all samples and thus sub-groups of reactions. The annotation element also replaces the elements used to describe DNA or RNA quality. Furthermore the DNA and RNA quantity elements, which were already modified in RDML version 1.1, were united as one quantity element in the current version. Some elements in RDML version 1.2 were added to provide further MIQE compatibility [7]. An example is the element “amplificationEfficiencySE” containing the uncertainty measure for the estimated PCR efficiency. For each target, this value results from the least-squares fit of the Cq versus log (input) observations of the standard curve or, alternatively, is calculated as the SEM of the observed efficiency values resulting from analysis of individual amplification curves [2, 8]. Finally, the documentation of several elements has been updated to clarify and avoid misinterpretation. This section highlights only the major changes; please check the online documentation as well as the supplemental data for a complete list of changes and the corresponding RDML standard definition (http://www.rdml.org). Despite the described changes, the majority of the elements are unmodified since RDML version 1.0 and therefore upgrading to version 1.2 should be no big burden for software developers.

### RDML-Ninja

**Fig. 1:**
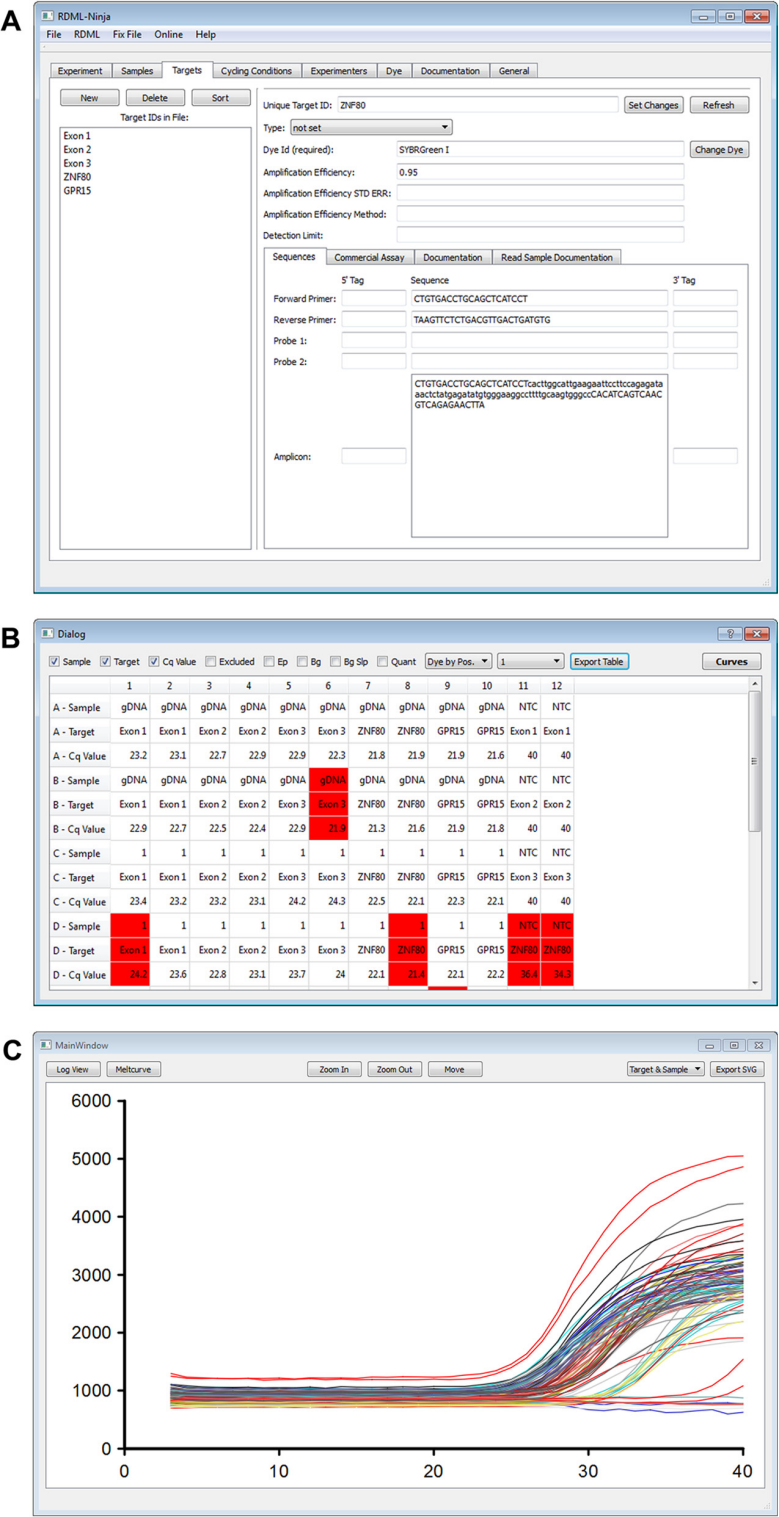
Open source editor RDML-Ninja. **a** Target information is displayed after selection of an ID in the left section. **b** The annotation and results of a single plate are visualized in an interactive table view. **c** Amplification results and melt point measurements can be plotted in a graph and exported in SVG format. A high resolution image is available as supplemental data

The RDML format is flexible due to many optional elements. On the one hand, this reduces the costs of implementation because it allows focusing on the parts of RDML required for the qPCR instrument functionality. On the other hand, no software is available today that can display all possible entries in an RDML file. Furthermore, the flexibility allowed some implementations to diverge from the intended use of RDML elements thus creating valid, but difficult to use, RDML files. RDML-Ninja was developed as reference implementation to fill this gap and serves to view and modify RDML files (http://sourceforge.net/projects/qpcr-ninja/). It provides access to all elements of an RDML file in an intuitive way (Fig. 1a-c). The majority of data can also be supplemented and/or modified, which enables the user to complement and correct the collected information. All such modifications have to be confirmed by the user by clicking a “set Changes” button (Fig. 1a). The content of some elements in an RDML file is restricted to pre-defined input. These restrictions are checked once the “set Changes” button is clicked and before the file is modified. However, the RDML consortium cannot foresee any reason to modify the raw fluorescence data collected by the instrument. Therefore, the entries of the “react” element cannot be modified by RDML-Ninja and are only displayed in a table view or in curve view (Fig. 1b, c). The table view can be exported as comma separated text files (CSV) that can be imported into other software packages such as Microsoft Office or other programs for analysis of amplification curves [8]. The curve view allows the export of graphics in the Scalable Vector Graphics format (SVG) for easy inclusion in presentations and papers. The SVG format can be modified by any vector graphics software without any loss of quality (e.g. Inkscape, http://www.inkscape.org).

In addition to the editor functionality, RDML-Ninja was enriched with RDML-specific functionality. An imported RDML file can be validated with the validator tool using the corresponding schema. The validator tool will state “validation successful” or provide information on the type of error that it has encountered. Currently, three versions of RDML exist and all were implemented into available qPCR systems and software. RDML-Ninja allows migrating files between all three versions at the cost of minor information loss if data were stored in elements not supported by the target version. This feature allows users to migrate their RDML data to the most recent version for further analysis when their instrument software supports only an older version.

### RDMLdb

**Fig. 2:**
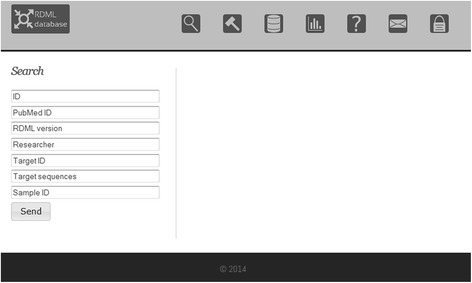
Search interface of RDMLdb. RDML files are found in the database based on query key elements

RDMLdb was designed for the exchange of RDML files (http://www.rdmldb.org). Users may upload their data and obtain a unique identifier (ID) referring to the uploaded file. This ID can be provided in publications and allows readers to extract the corresponding RDML file. Furthermore, users may add a PubMed ID to their files in order to link publications to the RDML files. The release of the file to the public can be delayed for up to one year to grant confidentiality during the reviewing and publication process. In addition to the ID, RDMLdb can be searched for PubMed ID, submitter name or RDML version (Fig. 2). RDML files can be downloaded from the website, either after searching for public files or using a direct link for private files in the review process. A statistics page keeps users informed on the number of records, linked publications, samples, targets and reactions stored in the database.

## Discussion

Over the past five years, the universal qPCR data exchange file format RDML has been well accepted by the scientific user community and is implemented in many qPCR instruments available today. Further, being part of the MIQE guidelines, it is endorsed by scientific journals and publishers. Although this is a big achievement, the use of RDML should not stop at this point. Currently, we see the bottleneck at the level of handling RDML files and RDML file exchange. The editor, RDML-Ninja, has been designed with different user types in mind. In the laboratory, RDML-Ninja should allow researchers to enter information into RDML elements not supported by the software of their qPCR instrument. Furthermore, RDML-Ninja can form a bridge between software supporting different RDML versions by handling the conversion. In the field of publishing, reviewers as well as readers can use RDML-Ninja to visualize and evaluate qPCR data in RDML files independent of instrument software. In the bioinformatics field, RDML-Ninja should assist software developers with the implementation of the RDML standard. Programmers can use RDML-Ninja to create files to challenge their software or to evaluate the validity of the files created by their software.

The online database RDMLdb will facilitate the storage and public exchange of RDML data. RDMLdb serves as a repository for RDML files where individual files are referred to by a unique ID. RDMLdb can thus function for qPCR data like the repositories for other gene expression measurement technologies, such as microarrays and RNA-sequencing.

RDML has the potential to connect all qPCR-data-associated processes in a lab. We envision users start by designing their qPCR assays using primer3plus and obtaining an RDML file containing the primer sequences. Then they extend the target information using RDML-Ninja with references to the gene ID and additional information. Many labs use a limited set of targets and qPCR cycling programs that could be collected in one comprehensive RDML file and shared among researchers. Once researchers start a qPCR run, they import this information from the lab file, enter the sample names and annotations, choose targets and edit the plate layout, adding targets and samples to each reaction in the run. After the real-time PCR instrument completes the qPCR run, it combines the run informations and the reactions and saves it into RDML files. Finally, researchers can analyze the raw data using the instrument software or the third party software of their choice and perform final statistical analysis. An RDML based pipeline is currently provided for RDML compatible qPCR instruments. The run, target and tissue information and the raw fluorescence data can be read into LinRegPCR [2]. This program performs qPCR quantification based on the analysis of the amplification curves and saves PCR efficiency values per target and Cq values per reaction back into the RDML file. When this RDML file is read into qbase + [3] the gene expression data can be normalized and further statistical analysis of the resulting relative expression levels can be performed. When, at the point of publication, the RDML file has been uploaded to RDMLdb and its ID is referred to in the publication the reviewers and readers can download the RDML file from the database, visualize the raw data as well as the derived efficiency and Cq values and thus review the complete data analysis process.

Ultimately, RDML should be extended to store all information required according to the MIQE guidelines. While the information required by MIQE may seem overwhelming to researchers, RDML offers an easy help to handle this information. All the information is entered only once and stored in a basic RDML file. Researchers would not have to re-enter this information with every qPCR run, but can instead import from this RDML file only the parts they need for the current qPCR run. Furthermore, integration of MIQE would allow checking to what extent MIQE information is provided by calculating the checklist completeness. This would serve researchers, enabling them to avoid the repetitive burden of manual documentation, as well as reviewers and readers, who require complete information to judge and replicate the published results.

## Conclusions

Our applications lower the barriers using RDML for data exchange. The open source editor RDML-Ninja allows visualisation of all RDML elements and migration between RDML versions. The database RDMLdb will serve as public online repository for RDML files. RDMLdb will ease data exchange between research groups and facilitate the use of RDML files in publications. Making qPCR data exchange more accessible will significantly enhance biology research, publication quality and qPCR data validation. With a tighter integration of MIQE a data format is in reach allowing collecting and exchanging all data required by MIQE at one.

### Availability and requirements

**Project name**: RDML, RDML-Ninja, RDMLdb

**Project home page**: http://www.rdml.org, http://sourceforge.net/projects/qpcr-ninja/, http://www.rdmldb.org

**Operating system(s)**: Platform independent

**Programming language**: Qt, C++, Perl, JavaScript, XML

**Other requirements**: Microsoft Windows 7 or higher, Macintosh OS X

**License**: GNU GPL

**Any restrictions to use by non-academics**: no

## References

[1] Lefever S, Hellemans J, Pattyn F, Przybylski DR, Taylor C, Geurts R, Untergasser A, Vandesompele J (2009). RDML: structured language and reporting guidelines for real-time quantitative PCR data. Nucleic Acids Res.

[2] Ruijter JM, Ramakers C, Hoogaars WM, Karlen Y, Bakker O, van den Hoff MJ and Moorman AF. Amplification efficiency: linking baseline and bias in the analysis of quantitative PCR data. Nucleic Acids Res. 2009; A37:e45. doi: 10.1093/nar/gkp045 10.1093/nar/gkp045 PMC2665230 19237396

[3] Hellemans J, Mortier G, De Paepe A, Speleman F, Vandesompele J (2007). qBase relative quantification framework and software for management and automated analysis of real-time quantitative PCR data. Genome Biol.

[4] Rödiger S, Burdukiewicz M, Blagodatskikh KA, Schierack P (2015). R as an Environment for the Reproducible Analysis of DNA Amplification Experiments. The R Journal.

[5] Pabinger S, Rödiger S, Kriegner A, Vierlinger K, Weinhäusel A (2014). A survey of tools for the analysis of quantitative PCR (qPCR) data. Biomolecular Detection and Quantification.

[6] Untergasser A, Cutcutache I, Koressaar T, Ye J, Faircloth BC, Remm M, Rozen SG (2012). Primer3–new capabilities and interfaces. Nucleic Acids Res.

[7] Bustin SA, Benes V, Garson JA, Hellemans J, Huggett J, Kubista M, Mueller R, Nolan T, Pfaffl MW, Shipley GL, Vandesompele J, Wittwer CT (2009). The MIQE guidelines: minimum information for publication of quantitative real-time PCR experiments. Clin Chem.

[8] Ruijter JM, Pfaffl MW, Zhao S, Spiess AN, Boggy G, Blom J, Rutledge RG, Sisti D, Lievens A, De Preter K, Derveaux S, Hellemans J, Vandesompele J (2013). Evaluation of qPCR curve analysis methods for reliable biomarker discovery: bias, resolution, precision, and implications. Methods.

